# R-LESS-RP versus C-LESS-RP: a single-institution retrospective comparative study

**DOI:** 10.1038/s41598-023-31021-z

**Published:** 2023-03-08

**Authors:** Yong Wei, Qianying Ji, Xin Zhou, Luming Shen, Xiaping Wang, Chen Zhu, Jian Su, Qingyi Zhu

**Affiliations:** 1 Department of Urology, The Second Affiliated Hospital of Nanjing Medical University, Nanjing, 210000 China; 2 Department of Urology, Jiangsu Province Hospital of Chinese Medicine, Affiliated Hospital of Nanjing University of Chinese Medicine, Nanjing, 210029 China; 3 Department of Oncology, First Affiliated Hospital of Nanjing Medical University, Nanjing, 210029 China; 4 Department of Pathology, The Second Affiliated Hospital of Nanjing Medical University, Nanjing, 210000 China

**Keywords:** Prostate, Prostate cancer

## Abstract

This study aimed to compare the peri- and postoperative outcomes of patients treated with conventional versus robot-assisted laparoendoscopic single-site radical prostatectomy (C-LESS-RP vs. R-LESS-RP). Data of patients with prostate cancer (106 who underwent C-LESS-RP, 124 underwent R-LESS-RP) were retrospectively collected and analyzed. All operations were performed by the same surgeon from January 8, 2018, to January 6, 2021, in the same institution. Information on clinical characteristics and perioperative outcomes was obtained from records at the medical institution. Postoperative outcomes were acquired from follow-up. Intergroup differences were retrospectively analyzed and compared. All patients had similar clinical characteristics in meaningful aspects. The perioperative outcomes were better with R-LESS-RP than with C-LESS-RP in terms of operation time (120 min vs. 150 min, p < 0.05), estimated blood loss (17.68 ml vs. 33.68 ml, p < 0.05), and analgesic duration (0 days vs. 1 days, p < 0.05). The drainage tube duration and postoperative stay did not differ significantly between groups. However, R-LESS-RP was more expensive than C-LESS-RP (56559.510 CNY vs. 44818.27 CNY, p < 0.05). The patients who underwent R-LESS-RP had better urinary incontinence recovery and higher European quality of life visual analog scale scores than those who underwent C-LESS-RP. However, no significant intergroup difference was noted in biochemical recurrence. In conclusion, R-LESS-RP could achieve better perioperative outcomes, especially for those skilled surgeons who have mastered C-LESS-RP. Additionally, R-LESS-RP accelerated the recovery from urinary incontinence effectively and presented some benefits in health-related quality of life with additional costs.

## Introduction

Prostate cancer (PCa) is the fourth most common cancer worldwide accounting for approximately 1.4 million patients estimated to be diagnosed according to the GLOBOCAN 2020^1^. Coincident with the widespread application of prostate specific antigen (PSA) testing, the incidence and early detection of preclinical PCa have increased^2^. In China, except for the broader utilization of diagnostic practices, a cumulatively westernized lifestyle and certain genetic polymorphism may collaboratively lead to the increase in the detection rate of PCa^3,4^. In the last 5 years, trends in incidence rates of PCa have remained stable in most Asian countries examined except an increasing trend observed in China^5^. Hence, although Chinese people are less affected by genetic inheritance than other races, the number of Chinese people diagnosed with PCa would continue to rise, especially in rural areas. Currently, the main treatments for PCa include radical prostatectomy, chemoradiotherapy and hormonal therapy, among which radical prostatectomy might serve as the only treatment strategy for treating the disease.

To date, laparoscopic radical prostatectomy has become the dominant treatment option for men with localized PCa. With the pursuit of minimally invasive treatment, many countries had a rapid adoption of conventional laparoendoscopic single-site radical prostatectomy (C-LESS-RP) or robot-assisted laparoendoscopic single-site radical prostatectomy (R-LESS-RP) such that the usage rate of robot assistance has increased to > 80% of all radical prostatectomies in the USA^6^. Although statistical data are lacking in China, increasing evident advantages have accelerated the rapid dissemination of R-LESS-RP in clinical practice.

Recently, several systematic reviews have suggested the superiority of robot-assisted radical prostatectomy (RARP) to open radical retropubic prostatectomy (ORP) in potency, urinary, positive surgical margin, biochemical recurrence (BCR), and complication rate^7–9^. By contrast, another study has reported that R-LESS-RP had similar outcomes in function to those of ORP and only provided benefits of a minimally invasive surgical approach to patients^10^.

The aforementioned studies mostly assessed and compared the benefits of RARP and ORP. However, few studies have compared the differences between RARP and laparoscopic radical prostatectomy (LRP), let alone the comparative study of C-LESS-RP and R-LESS-RP. A LAP-01 multicenter, patient-blinded, randomized controlled trial has demonstrated that the urinary incontinence recovery among patients with RARP was superior to patients with LRP after 3 months, although this difference diminished at the 12-month follow-up^11^. A prospective randomized trial has revealed significant differences in continence between patients who underwent RARP and those who underwent LRP 12 months postoperatively^12^. In total, 120 patients with PCa who received LRP demonstrated statistically significant differences 12 months postoperatively following RARP. However, the benefits of R-LESS-RP remain unclear despite having an increased surgery cost. For patients, the extra costs for R-LESS-RP might lead to their unwillingness to select such method. Meanwhile, because of the increased technical difficulty of R-LESS-RP, it failed to gain popularity among urologists. Moreover, whether this operating technique could achieve a high-level of evidence in perioperative and function outcomes was still unproven. Hence, we conducted a retrospective study to compare the differences in peri- and postoperative outcomes of patients with PCa treated with C-LESS-RP and R-LESS-RP.

## Materials and methods

### Patients

**Figure 1:**
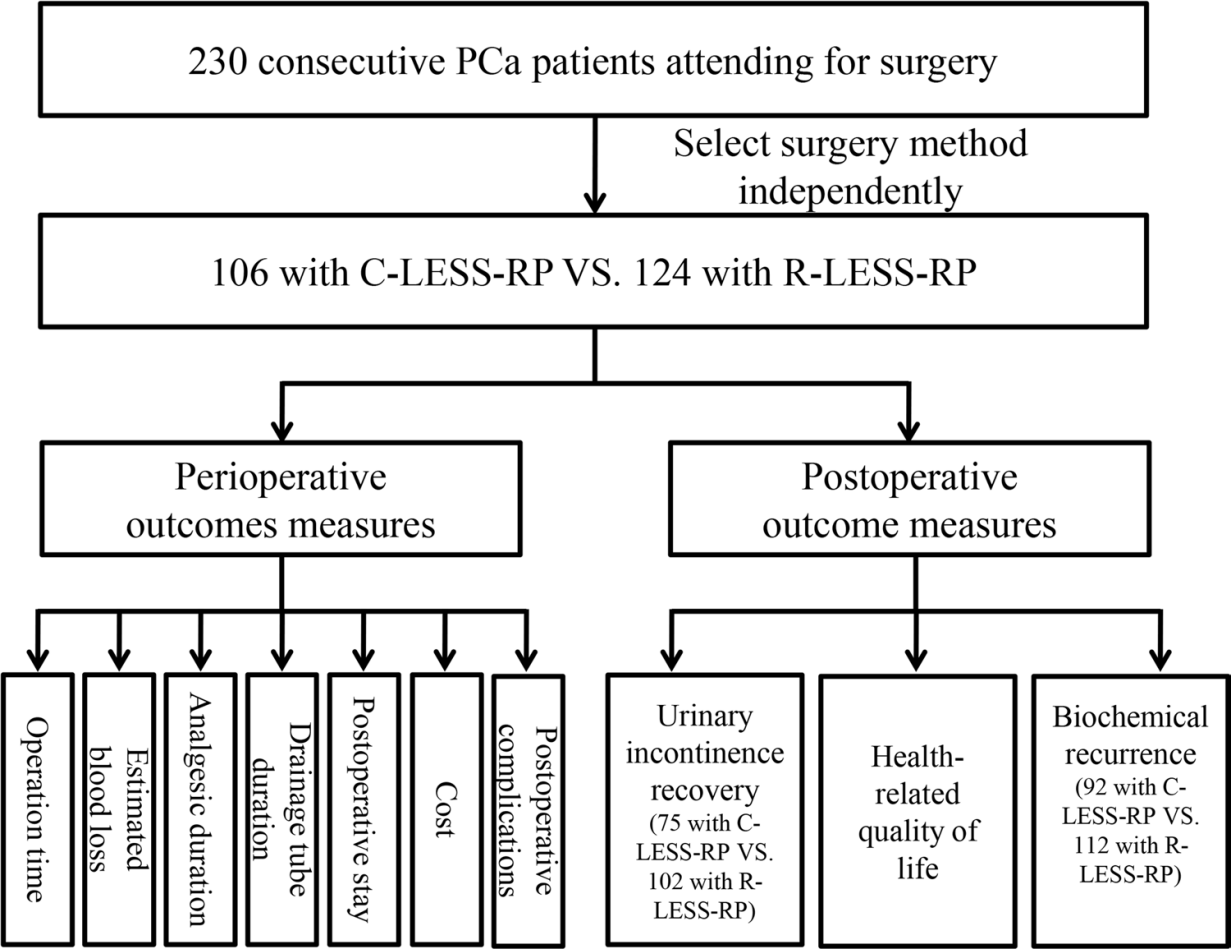
The flow chart of the study.

A consecutive cohort of PCa patients attending for surgery treatment was recruited thus limiting bias. Altogether, 230 patients who underwent single-site LRP performed by the same surgeon and pathologically diagnosed with PCa between January 8, 2018 and January 6, 2021 at Jiangsu Province Hospital of Chinese Medicine were enrolled in our study. The process, cost, risk, advantages, and disadvantages of the two surgical methods were discussed with the patients preoperatively. After detailed communication with patients, the surgical option for LESS-RP or R-LESS-RP was independently selected by each patient preoperatively. In total, 106 patients selected C-LESS-RP and 124 underwent R-LESS-RP with the Da Vinci IV robotic platform. Informed consent was obtained from all the participants. Data on sociodemographic and clinical characteristics including age, body mass index (BMI), PSA level, prostate volume, Gleason score, and the eighth edition American Joint Committee on Cancer tumor-nodes-metastasis (TNM) staging were retrieved from medical records. This study was approved by the Ethics Committee of the Affiliated Hospital of Nanjing University of Chinese Medicine (Institutional Ethics Committee ID: 2020NL-KS051). All procedures were conducted in accordance with relevant guidelines and regulations. The STROBE checklist of the study was presented in Table S1. The flow diagram for the study was shown in Fig. 1.

### Perioperative outcomes measures

Perioperative data including operation time, estimated blood loss (EBL), analgesic duration, drainage tube duration, postoperative stay, and complications of all the patients were obtained from the institution’s health information system. The operation time referred to the duration of skin incision to skin suture. In R-LESS-RP, the operation time included the setup time. Considering that an analgesia pump is not routinely used in single-site surgery, the analgesic duration was defined as the number of days of postoperative use of analgesics which were mainly administered intravenously once a day. Additionally, information on total hospitalization costs of all the patients was also collected from the health information system.

### Postoperative outcome measures

Postoperative outcomes including urinary incontinence and postoperative serum PSA levels and subsequent therapy were followed up regularly. Urinary incontinence recovery was evaluated according to the number of pads used daily, and those who used 0–1 pad daily were considered to have recovered from urinary incontinence^13^. Postoperative PSA levels were used to monitor for BCR, which was defined as postoperative PSA level of ≥ 0.2 ng/ml on two consecutive occasions during follow-up^14^. Additionally, a 5-level version of the European quality of life five-dimensional descriptive system (EQ-5D-5L) and EQ-VAS was used to evaluate whether the patients’ postoperative health-related quality of life after surgeries differed after the two surgical approaches^15,16^. EQ-5D-5L is an instrument comprising five dimensions (mobility, self-care, usual activities, pain or discomfort, and anxiety or depression). Each domain is divided into five levels of severity (none, slight, moderate, severe, extreme problems, or unable to). Patients were asked to rate their self-perceived health state from 0 (worst health) to 100 (best health) in the EQ-VAS^14,17^.

### Statistical analyses

Data were analyzed using the SPSS 26.0 or R software (version 4.1.0). Group differences were assessed using t-test (normal distribution data) or Mann–Whitney test for continuous variables and χ^2^ or Fisher’s exact test for categorical variables at follow-up timepoints. BCR and urinary incontinence were estimated using Kaplan–Meier analysis and were compared using log-rank test. A two-sided p < 0.05 was considered statistically significant.

## Results

### Clinical characteristics

Altogether, 230 patients with PCa were enrolled in our study. After detailed communication with the patients, the surgical option was selected by the patients independently. On the principle of voluntary selection, 106 patients selected C-LESS-RP and 124 selected R-LESS-RP as their surgical modalities. As demonstrated in Table 1, the patients in the two groups had similar clinicopathologic data. At baseline, patient sociodemographic and clinical characteristics such as age, BMI, prostate volume, grade, and clinical stage were not significantly different between the two groups. However, the average preoperative PSA of patients who underwent C-LESS-RP was higher than those who underwent R-LESS-RP.

**Table 1:** Clinical characteristics of patients enrolled in the study (χ^2^ or Fisher’s exact test for categorical and t-test or Mann–Whitney test for continuous variables).

	C-LESS-RP	R-LESS-RP	p value
Number	106	124	
Age (years) (median ± SD)	71 ± 6.93	70 ± 8.01	0.63
BMI (median ± SD)	23.4 ± 3.10	23.7 ± 2.86	0.81
Preop tPSA (ng/ml) (median ± SD)	15.67 ± 41.27	11.7 ± 36.73	0.037
Prostate volume (ml) (median ± SD)	38.58 ± 23.21	41.5 ± 29.31	0.32
Pathology Gleason score, n (%)			
3 + 3	9 (8.82)	17 (13.71)	0.07
3 + 4	17 (16.04)	33 (26.61)	
3 + 5	1 (0.94)	1 (0.81)	
4 + 3	18 (16.98)	27 (21.78)	
4 + 4	17 (16.04)	13 (10.48)	
4 + 5	25 (23.58)	13 (10.48)	
5 + 4	7 (6.60)	10 (8.06)	
5 + 5	1 (0.94)	2 (1.61)	
Unknown	11 (10.38)	7 (5.64)	
Clinical stage, n (%)			
I	8 (7.55)	9 (7.26)	0.45
II	27 (25.47)	45 (36.29)	
III	49 (46.23)	51 (41.13)	
IV	20 (18.87)	18 (14.52)	
Unknown	2 (1.89)	1 (0.81)	

*C-LESS-RP* conventional laparoendoscopic single-site radical prostatectomy, *R-LESS-RP* Robot-assisted laparoendoscopic single-site radical prostatectomy, *BMI* body mass index, *SD* standard deviation.

### Perioperative outcomes

**Figure 2:**
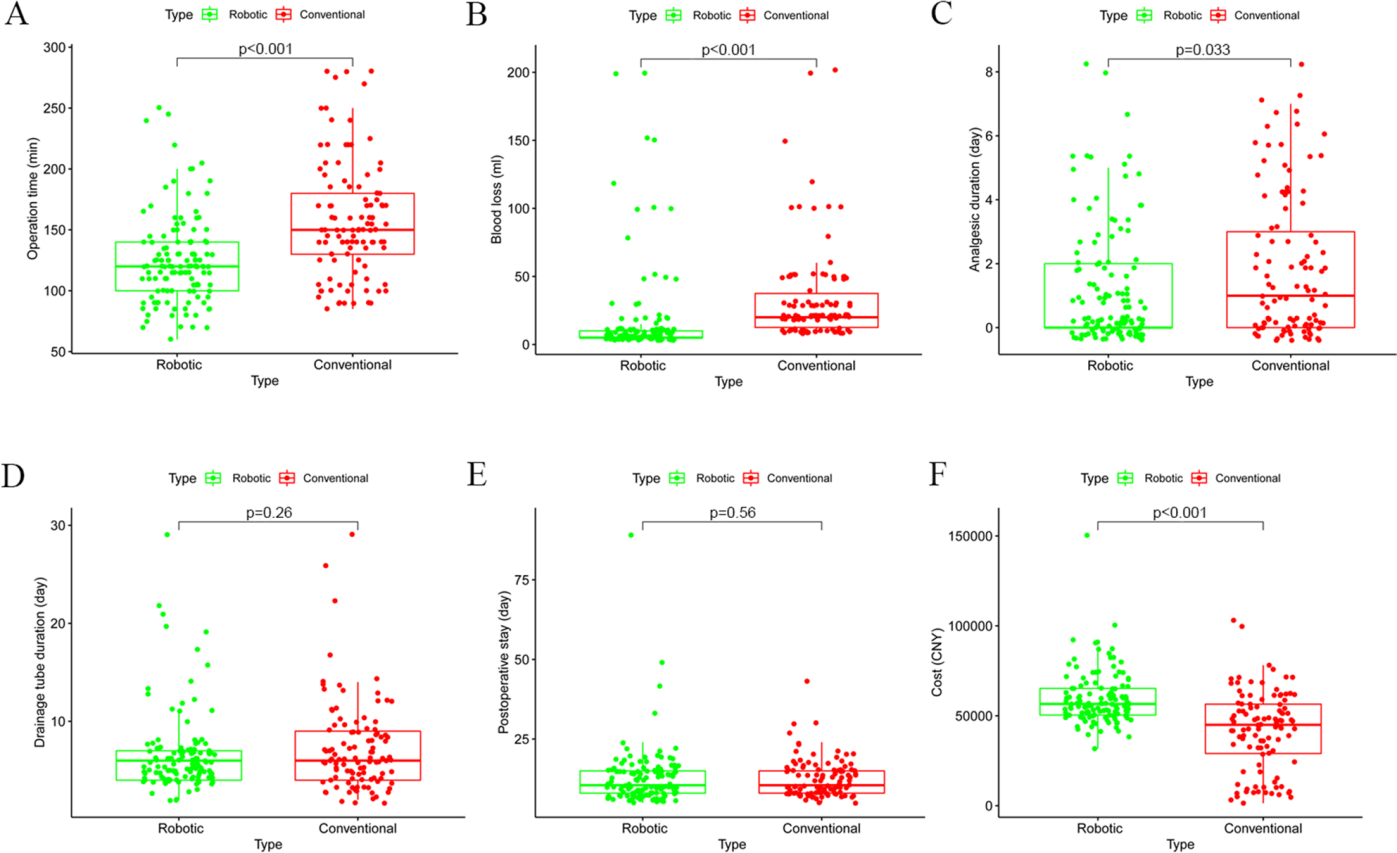
Comparison of perioperative outcomes between patients with the two different surgery approaches ((**A**) operation time; (**B**) blood loss; (**C**) analgesic duration; (**D**) drainage tube duration; (**E**) postoperative stay; (**F**) total hospitalization costs). According to t-test or Mann–Whitney test.

To compare the advantages and disadvantages of the two surgical methods, we mainly evaluated the perioperative data (Fig. 2 and Table 2). For operation time, the surgeon took longer time to complete C-LESS-RP than R-LESS-RP. The median operation time of R-LESS-RP was 120 ± 36.29 min, whereas that of C-LESS-RP was 150 ± 47.39 min (p < 0.01). It is worth mentioning that the average standby time for R-LESS-RP was 14.6 ± 3.2 min. Consistent with the operation time, a distinct difference of EBL during operations between two surgical approaches was identified (p < 0.01). The median EBL of R-LESS-RP was 17.68 ± 34.29 ml, whereas that of C-LESS-RP was 33.68 ± 38.81 ml. No patient required an intraoperative or postoperative blood transfusion in either group. Additionally, the analgesic duration of patients who underwent R-LESS-RP (0 ± 1.8 days) was apparently less than those of another surgical approach (1 ± 2.23 days, p = 0.033). Nevertheless, the drainage tube duration and postoperative stay did not significantly differ between the two surgical approaches. As predicted, total hospitalization costs were markedly different between the two surgical approaches. The median hospitalization cost of patients who underwent R-LESS-RP was 56,559.5 ± 14,991.99 CNY. Meanwhile, those who underwent C-LESS-RP spent a lower median cost of 44,818.27 ± 22,041.35 CNY (p < 0.01).

**Table 2:** Perioperative outcomes of patients with C-LESS-RP and R-LESS-RP (t-test or Mann–Whitney test).

	C-LESS-RP	R-LESS-RP	p Value
Operation time (min) (median ± SD)	150 ± 47.39	120 ± 36.29	< 0.01
Estimated blood loss (ml) (median ± SD)	20 ± 38.81	5 ± 34.29	< 0.01
Analgesic duration (days) (median ± SD)	1 ± 2.23	0 ± 1.8	0.033
Drainage tube duration (days) (median ± SD)	6 ± 4.55	6 ± 4.15	0.26
Postoperative stay (days) (median ± SD)	10.5 ± 5.98	10.5 ± 9.48	0.56
Cost (CNY) (median ± SD)	44,818.27 ± 22,041.35	56,559.51 ± 14,991.99	< 0.01

*C-LESS-RP* conventional laparoendoscopic single-site radical prostatectomy, *R-LESS-RP* Robot-assisted laparoendoscopic single-site radical prostatectomy, *SD* standard deviation.

Additionally, postoperative complications were counted. As presented in Table 3, 16 patients experienced postoperative complications (seven who underwent C-LESS-RP versus nine who underwent R-LESS-RP). Five patients developed urinary fistula after C-LESS-RP, and six patients experienced the same situation with R-LESS-RP, although no significant intergroup difference was noted. One patient who underwent C-LESS-RP had a postoperative ileus that was treated using a second surgery. Two patients who underwent R-LESS-RP developed postoperative ileus; only one case was spontaneously resolved, and the other patient received a second surgery. One patient had urethral stenosis after R-LESS-RP. Besides, one patient in each group had chylous leakage. Most of the complications were minor, except for one case of postoperative ileus in each group.

**Table 3:** Number of patients developed complications after the two surgery approaches (χ^2^ or Fisher’s exact test).

	C-LESS-RP	R-LESS-RP	p value
Urinary fistula, n (%)	5 (4.71)	6 (4.83)	0.996
Postoperative ileus, n (%)	1 (0.94)	2 (1.61)	0.656
Urethral stenosis, n (%)	0	1 (0.81)	0.354
Chylous leakage, n (%)	1 (0.94)	1 (0.81)	0.911

*C-LESS-RP* conventional laparoendoscopic single-site radical prostatectomy, *R-LESS-RP* Robot-assisted laparoendoscopic single-site radical prostatectomy.

### Postoperative outcomes

#### Urinary incontinence recovery

**Figure 3:**
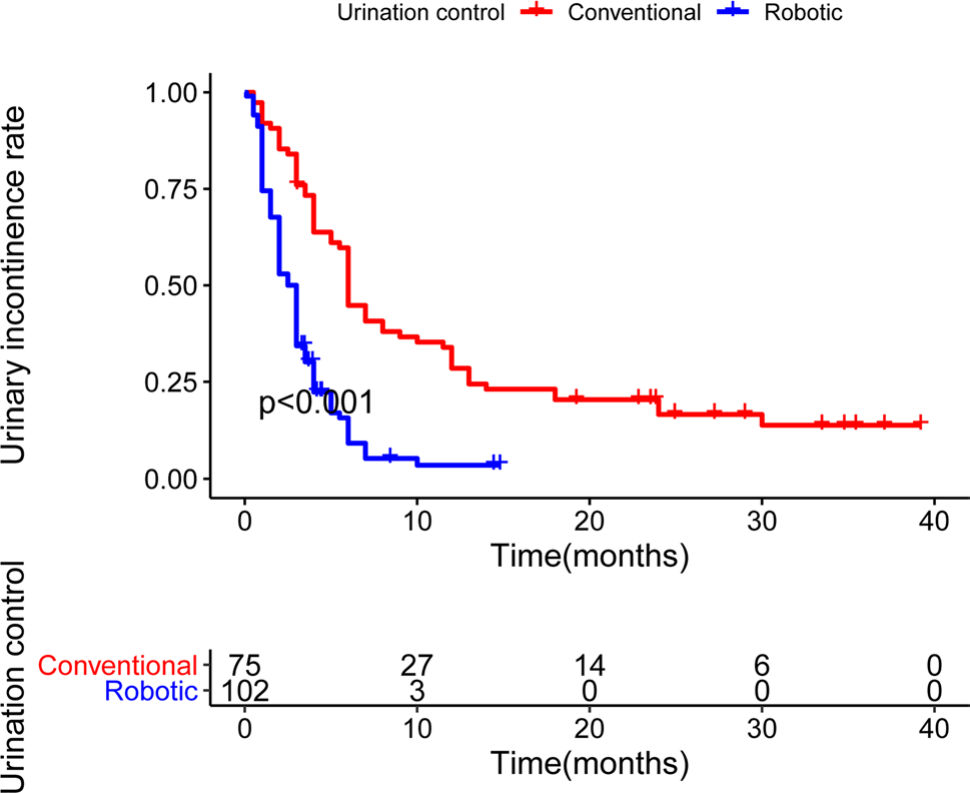
Difference in urinary incontinence recovery after operations with the two surgery approaches estimated with KM curves. According to the Kaplan–Meier methodology and compared using log-rank test.

Regardless of the type of operation received, the patients received the same postoperative care, and no additional rehabilitation training was performed. Anti-infection, pain relief, and other symptomatic treatment were provided postoperatively during hospitalization. Significant differences in duration to achieve urinary incontinence recovery following the operations were observed between the two surgical approaches. After excluding patients who were lost to follow-up, the data of 75 patients who underwent C-LESS-RP and 102 patients who underwent R-LESS-RP were retrospectively analyzed. None of the cases were severe that required surgery as a treatment method. The results suggested that the incontinence rate after R-LESS-RP was higher than that after C-LESS-RP. The patients who underwent R-LESS-RP significantly spent less time to achieve satisfactory urinary function (Fig. 3).

### Health-related quality of life

**Figure 4:**
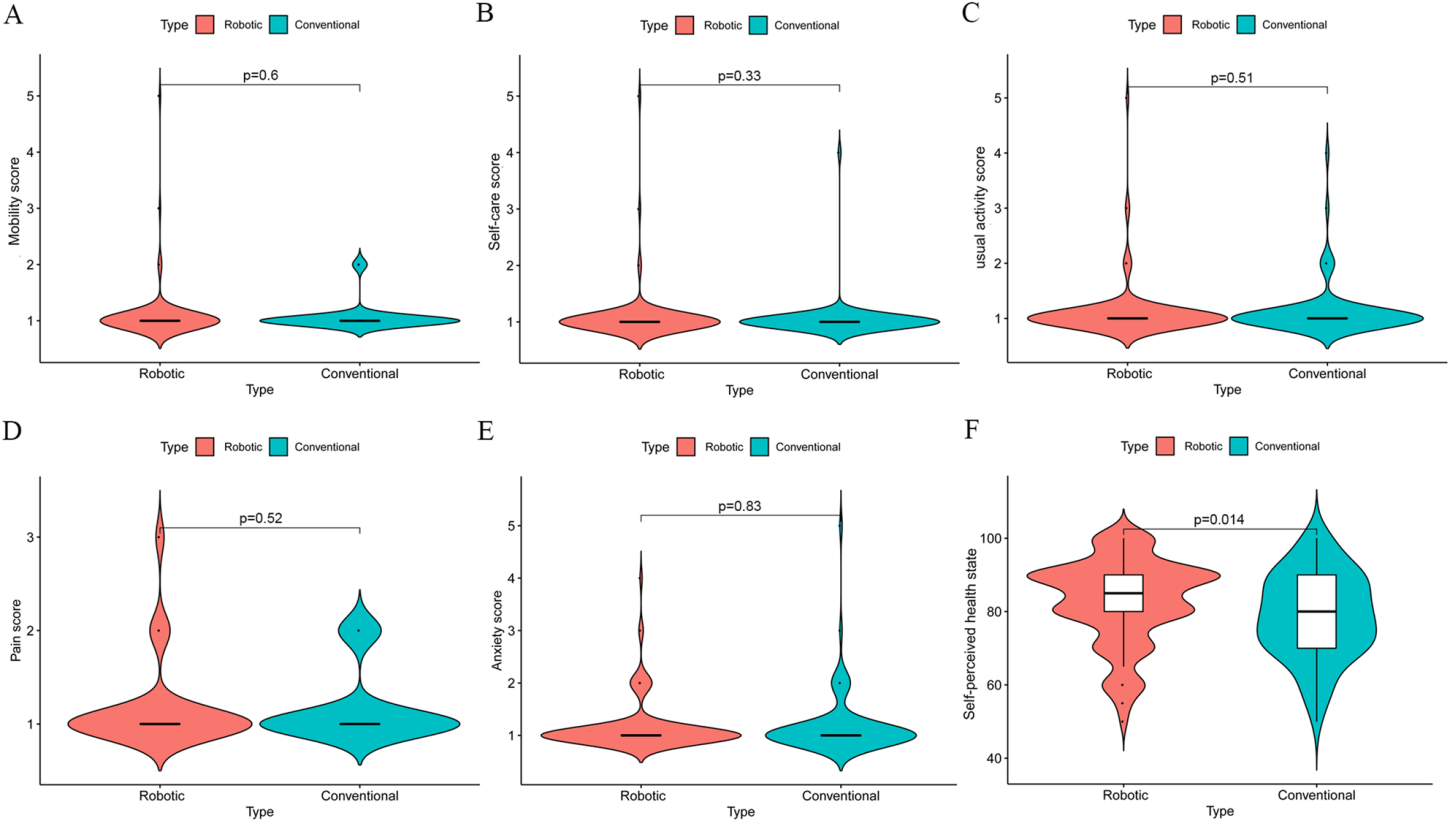
Comparison of postoperative health-related quality of life in patients with R-LESS-RP to those underwent C-LESS-RP ((**A**) mobility; (**B**) self-care; (**C**) usual activity; (**D**) pain; (**E**) anxiety; (**F**) self-perceived health state). According to t-test or Mann–Whitney test.

To access the postoperative health-related quality of life, EQ-5D-5L and EQ-VAS questionnaires were used to evaluate six aspects. The results of the EQ-5D-5L revealed no difference in the five aspects of mobility, self-care, usual activities, pain/discomfort, and anxiety/depression (Fig. 4A–E). The pain score did not differ between the two groups (p = 0.52). However, self-perceived health state was significantly better for patients following R-LESS-RP than for those who underwent C-LESS-RP (82.88 ± 11.58 vs. 78.84 ± 11.58, p = 0.014; Fig. 4F).

### Biochemical recurrence

**Figure 5:**
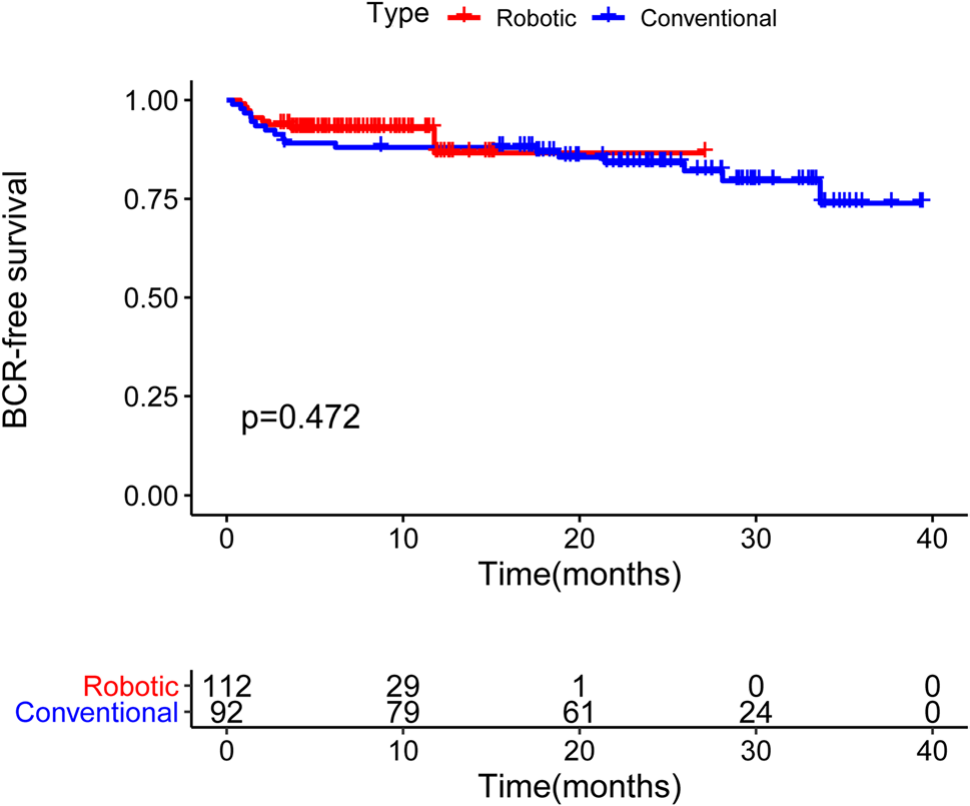
BCR survival of PCa patients according to the different surgery approaches. *BCR* biochemical recurrence. According to the Kaplan–Meier methodology and compared using log-rank test.

Altogether, 204 patients were followed up to assess the effect of different surgery methods on BCR (92 underwent LESS-RP, 112 underwent R-LESS-RP). The data were composited PSA results of patients’ report during follow-up and records from medical situation. However, it revealed no evidence of interaction between surgical approaches and BCR after surgery (Fig. 5).

## Discussion

Robot-assisted technology could facilitate the accomplishment of some important surgical steps of the procedure^18^. Since Binder has reported the first RARP in 2001, offering technical innovations including three-dimensional visualization, articulated instruments, and tremor filtration, it has immediately become popular among urologists^19^. The Da Vinci Xi surgical system released in 2014 has smaller and lighter operating arms, and the suspended mobile platform can allow adjusting the operating direction freely, thereby expanding the operative field exposure. Robotic surgery has gained increasing attention because of its advantages of clearer vision, more flexible operation angle, and more precise exposure anatomy. Surgeons advocating single-site surgery may prefer the Da Vinci single-port robotic system because of the principle of anterior pelvic support preservation and its touted early continence recovery^20^. However, whether the extra cost is worth it remains controversial.

This retrospective comparative analysis demonstrated the feasibility and preponderance of R-LESS-RP using the Da Vinci Xi system. The EBL and operation time were important for assessing a new surgical technique. The median operation time of R-LESS-RP was only 120 min (vs. 150 min for C-LESS-RP, p < 0.05). Slightly different from that of the previous study^21^, the EBL was less in the R-LESS-RP procedure than in the other prostatectomy procedure (17.68 ml vs. 33.68 ml, p < 0.05). As the EBL might be positively associated with the operation time, the surgeon could complete the surgery more quickly with less blood loss. The stabilizer of the Da Vinci system also helped to complete the surgeries more quickly with less blood loss. Moreover, as R-LESS-RP is minimally invasive, this could be the reason for the shorter analgesic duration (0 days vs. 1 days, p < 0.05) associated with it. The shorter analgesic duration might help with faster postoperative recovery. Otherwise, the results revealed that robot-assisted surgery did not reduce or increase the incidence of complications. The drainage tube duration and postoperative stay were not significantly different between the two surgical approaches. As expected, the hospitalization cost of patients who underwent R-LESS-RP was more expensive than that of the patients who underwent C-LESS-RP (44,818.27 CNY vs. 56,559.51 CNY, p < 0.05). Nevertheless, the difference in the cost was smaller than expected.

After prostatectomy, urinary incontinence recovery is one of a patient’s major concerns regardless of the type of surgical procedure performed. Because of the facilitated frozen section analysis, R-LESS-RP was considered a better approach for sparing the neurovascular bundle. Neurovascular bundle preservation was performed depending on the individual surgeon’s discretion based on clinical staging. A mature laparoscopic radical prostatectomy series revealed that the 12-month urinary continence recovery was 66–95%^22^. Another study has reported that the prevalence of urinary incontinence after RARP was 4–31%^23^. According to the statistical data of our institution, the 12-month continence rate after R-LESS-RP was higher than that after C-LESS-RP (74% vs. 50%, Table 4). A significant difference in urinary incontinent recovery was identified between both groups. The time of urinary incontinence recovery of patients who underwent R-LESS-RP was faster than that of patients who underwent C-LESS-RP. The traditional single-site laparoscopic surgery often lacks the “triangle” relationship of surgical operation, special instruments need to be used during the operation, and the operation space is relatively limited. The mutual interference between the lens and other instruments greatly increases the difficulty of the operation. The learning curve of the surgeon is long, especially in the aspects of exposure and suture. The advantages of the robot-assisted surgery system are reflected in its “wrist” device significantly expanding the scope of surgery, and its unique motion scaling and vibration control that effectively improves the flexibility and accuracy of surgery.

**Table 4:** Urinary incontinence recovery rate for PCa patients after operations (χ^2^ test).

	C-LESS-RP	R-LESS-RP	p value
3-mo continence rate	17%	54%	< 0.01
6-mo continence rate	29%	70%	< 0.01
12-mo continence rate	50%	74%	< 0.01

*C-LESS-RP* conventional laparoendoscopic single-site radical prostatectomy, *R-LESS-RP* Robot-assisted laparoendoscopic single-site radical prostatectomy.

The data of neurovascular bundle tissue sparing on histology were lacking in both groups in this study. However, the preoperative PSA of the patients who underwent C-LESS-RP was higher, which might affect neurovascular bundle tissue sparing and urinary incontinence recovery. Furthermore, although erectile function is also a postoperative concern for patients, we did not analyze such data. As erectile function can continue to improve for up to 3 years postoperatively^24^, any difference in outcomes between these surgical techniques might not become apparent for some time.

According to the outcomes assessed using the self-reported EQ-5D-5L questionnaire, the EQ-VAS score was certainly connected with different surgical approaches (82.88 for patients who underwent R-LESS-RP vs. 78.84 for patients who underwent C-LESS-RP). Such outcomes might also be related to self-psychological status, urinary continence recovery, and advances in oncology which require further analysis. Different surgical approaches did not improve the patients’ health-related quality of life (mobility, self-care, usual activities, pain or discomfort, anxiety, or depression) postoperatively.

A previous study has demonstrated the lack of high quality evidence to support superiority of oncological outcomes, not only prostate cancer-specific survival in particular, but also biochemical and recurrence-free survival^25^. Similarly, we did not observe any short-term intergroup difference in biochemical recurrence. The dropout ratio of urinary incontinence recovery data for the patients who underwent C-LESS-RP was higher than for those who underwent R-LESS-RP (p = 0.039). For biochemical recurrence, no significant intergroup difference in dropout ratio was observed (p = 0.4). For each patient enrolled in our study, we all similarly followed the postoperative outcomes regardless of the specific surgery received by the patient. However, the patients who underwent R-LESS-RP were more likely to pay attention to their recovery status and share their information. Some patients also provided equivocal information that was excluded in our study. Some patients also changed or provided wrong contact information. For biochemical recurrence data, we definitely identified the PSA value from the patients from our hospital or presentation from local hospital for some patients. These might explain the differences in the dropout ratio between the two clinical outcomes. Although a slightly better trend was observed, further long-term clinical research is necessary.

Previously, the LAP-01 trial conducted by researchers from the University of Leipzig and other institutions in Germany proved that RARP was superior to the traditional LRP in terms of improving urinary continence after 3 months^11^. It is generally believed that patients who underwent RARP can continue to recover after 3 months, especially in terms of urinary continence. Therefore, functional and oncological data for 12 months are crucial for comparing the results of different surgical methods. In the former trial, further comparison on the urinary continence and oncology results between RARP and LRP during the 12-month follow-up indicated no significant difference. Similar to our outcomes, the postoperative recovery of urine continence in the short term was faster among patients after robot-assisted surgery. However, more differences between the two surgical methods were noted, which may be due to the high difficulty of traditional single-site laparoscopic surgery. Moreover, the application of robot-assisted technology can greatly reduce the difficulty of operation.

It is important to emphasize that all the operations were performed by the same surgeon who has engaged in urological single-port surgery for 10 years. As a surgeon who had mastered C-LESS-RP, robot-assisted technology could contribute to reserve energy and simultaneously achieve better perioperative outcomes.

In all the cases, R-LESS-RP demonstrated evident advantages of perioperative outcomes for patients with PCa and was conducive for recovery from urinary incontinence postoperatively. With the development of technology and economy, more patients would be willing to select robotic surgery.

## Limitation

One limitation of this study is selection and treatment bias. The treatment method is mainly determined by the patient, and the different economic status among patients may have influenced postoperative recovery. Another limitation was that only one surgeon performed the surgeries as the study was conducted in a single institution study. Thus, these results might not be generalized to other settings. A prospective randomized trial with a larger sample size for repeated validation is required in the future.

## Conclusions

R-LESS-RP had the following perioperative advantages: shorter operation time, less blood loss, and shorter analgesic duration. The recovery of urinary incontinence was significantly faster for patients who underwent R-LESS-RP than for those who underwent C-LESS-RP. The relatively extra cost remained a barrier to the popularity of R-LESS-RP which could be solved gradually.

## Supplementary Information

Supplementary Table S1.

## Data Availability

All data generated or analyzed during this study are included in this published article.
